# Deep Source Localization with Magnetoencephalography Based on Sensor Array Decomposition and Beamforming

**DOI:** 10.3390/s17081860

**Published:** 2017-08-11

**Authors:** Yegang Hu, Yicong Lin, Baoshan Yang, Guangrui Tang, Tao Liu, Yuping Wang, Jicong Zhang

**Affiliations:** 1 School of Biological Science and Medical Engineering, Beihang University, Beijing 100191, China; huyegang0630@126.com (Y.H.); bshyang@buaa.edu.cn (B.Y.); tangguangrui@buaa.edu.cn (G.T.); tao.liu@buaa.edu.cn (T.L.); 2 Beijing Advanced Innovation Center for Biomedical Engineering, Beihang University, Beijing 100191, China; 3 Beijing Advanced Innovation Center for Big Data-Based Precision Medicine, Beihang University, Beijing 100191, China; 4 Department of Neurology, Xuanwu Hospital, Capital Medical University, Beijing 100053, China; liny@xwhosp.org (Y.L.); wangyuping@xwhosp.org (Y.W.); 5 Brain Functional Disease and Neuromodulation of Beijing Key Laboratory, Beijing 100053, China

**Keywords:** magnetoencephalography, deep source localization, iterative matrix decomposition, beamforming, epileptogenic zone, mesial temporal lobe epilepsy

## Abstract

In recent years, the source localization technique of magnetoencephalography (MEG) has played a prominent role in cognitive neuroscience and in the diagnosis and treatment of neurological and psychological disorders. However, locating deep brain activities such as in the mesial temporal structures, especially in preoperative evaluation of epilepsy patients, may be more challenging. In this work we have proposed a modified beamforming approach for finding deep sources. First, an iterative spatiotemporal signal decomposition was employed for reconstructing the sensor arrays, which could characterize the intrinsic discriminant features for interpreting sensor signals. Next, a sensor covariance matrix was estimated under the new reconstructed space. Then, a well-known vector beamforming approach, which was a linearly constraint minimum variance (LCMV) approach, was applied to compute the solution for the inverse problem. It can be shown that the proposed source localization approach can give better localization accuracy than two other commonly-used beamforming methods (LCMV, MUSIC) in simulated MEG measurements generated with deep sources. Further, we applied the proposed approach to real MEG data recorded from ten patients with medically-refractory mesial temporal lobe epilepsy (mTLE) for finding epileptogenic zone(s), and there was a good agreement between those findings by the proposed approach and the clinical comprehensive results.

## 1. Introduction

MEG is a functional neuroimaging technique that captures neural activity with high spatiotemporal resolution and minor signal deterioration from the skull and scalp [1,2,3,4]. Since different source activities can generate an identical magnetic field distribution at the MEG sensor arrays, source localization techniques become an essential step for finding real sources by modeling the inverse solution in one assumption. At present, many studies have shown that MEG source localization methods seem to be effective and helpful for detecting surface sources, especially for finding the epileptogenic zone of neocortical epilepsy [5,6]. Meanwhile, deep source detection plays a more and more important role in clinical applications, such as finding the real discharge region with mTLE patients. However, the ability about detecting deep sources, such as those in the mesial temporal lobe, remains in question [7,8,9]. Although the magnetometer has been considered for spike detection in patients with mesial temporal epileptic focus [10], the clinical utility of MEG has not been recognized by consensus in preoperative evaluation with epileptogenic foci in deep regions [11]. Thus, a technical challenge is whether signals from deep sources could be well detected and correctly located using a MEG device and source localization algorithms.

Until now, many source localization algorithms [12,13,14] have been proposed for seeking real brain activity locations, wherein beamforming techniques [15] comprise a well-known method family and plays a key role in signal processing and neuroimaging [16]. The characteristic of those methodologies is spatial filtering that the MEG sensor signals are filtered by different beams based on lead-field vectors corresponding to specific source-grid points [17]. Since the projection vector is obtained by minimizing the filter output power subject to a linear constraint, LCMV beamforming (also called vector beamforming) is a trustworthy spatial filter. Nevertheless, existing vector beamformers have been limited to estimate the sensor covariance matrix based on the original sensor arrays and, thus, are sensitive to noise levels and irrespective of the intrinsic feature in the sensor’s space.

Matrix decomposition or factorization can represent the original space by a lower-dimensional approximation, and has been successfully applied in various research fields, such as model reduction, feature extraction, classification, clustering and blind source separation, etc. [18]. MEG sensor measurements are typical spatiotemporal signals [19], and the data matrix is constructed by MEG measurements during one time window. Meanwhile, different rows of this matrix denote different sensors, and different columns denote different time points in a time series of MEG measurements that are generated by neuronal activities. However, these MEG sensor recordings are correlated both in spatial sensors and in temporal information. The major challenge is to remove the redundant information and extract the intrinsic feature via a matrix decomposition technique [20]. Herein, sensor array decomposition and beamforming techniques would be adopted for finding deep sources.

In this manuscript, we proposed a new source localization algorithm for detecting deep brain sources in MEG measurements, which is a derived version of the vector beamforming approach. First, matrix decomposition technique based on an iterative process [21,22] was employed for reconstructing the sensor arrays, which could characterize the intrinsic discriminant feature. Next, the covariance matrix was estimated under the newly-reconstructed space, and a minimum variance method was used to approximate the weight vector under linear constrains for finding deep brain activities. Then, the proposed modified beamforming approach was verified using simulated MEG data generated with deep sources, and was compared with two other common methods (MUSIC [12] and LCMV [15]). Taking into account that conventional beamformer techniques are successful in localizing neuronal sources that are uncorrelated, we assumed only one single source was found in the following simulated data and epilepsy patients.

In addition, we further assessed the performance of the proposed approach using real data from mTLE patients. As is known, mTLE is a type of epilepsy with deep source origins and plays an important role in refractory focal epilepsy [23,24]. Around two thirds of patients with temporal lobe epilepsy could achieve seizure freedom via resective epilepsy surgery, and MEG examination results become a key role in preoperative evaluation with the epileptogenic zone and in guiding surgical placement of intracranial electrodes [25,26]. Thus, we applied the proposed procedure to MEG data to find the epileptogenic zone in medically-refractory mTLE, and compared the results of it with the Signal Processor Software (SPS) method (Elekta Neuromag, version 2.94) [27], which is clinically acknowledged and very commonly used for localizing epileptogenic zone in clinical practice.

The rest of this paper is organized as follows: related background introduction is covered in Section 1. The deep source localization algorithm based on sensor decomposition and beamforming is described in Section 2. The details of experimental data and results are provided in Section 3. The paper is finally concluded in Section 4.

## 2. Methods

### 2.1. Iterative Matrix Decomposition

For observed signal matrix **X** = [***x***_1_, ***x***_2_, ..., ***x****_M_*], which is acquired from MEG sensors, the inverse solution model is given as:

**X** = **LD** + **ε** (1)

where ***x****_i_* is an *N* × 1 vector of the MEG measurements at *i*-th time point, *N* is the number of MEG sensors, **L** is the *N* × *J* (lead-field) gain matrix, *J* denotes the number of unknown dipole moment parameters, **D** is a *J* × *M* dipole moment matrix for given time series, and **ε** denotes the *N* × *M* noise matrix. In fact, how to compute the solution **D** plays a prominent role in source localization according to the known signal matrix **X**. Since the space of the signal matrix X may be disturbed by noises and other factors, it will result in inaccurate localization results. Especially, the deep brain signals received by the sensors are very weak, and are more sensitive to those noises. In order to localize the deep sources accurately, it is necessary to remove the signal interference without distortion. However, most of the existing localization algorithms are proposed by using the original signal matrix. At present, signal reconstruction based on matrix decomposition can restore the cleaner signal, and has been successfully applied in many fields [28].

**Figure 1:**
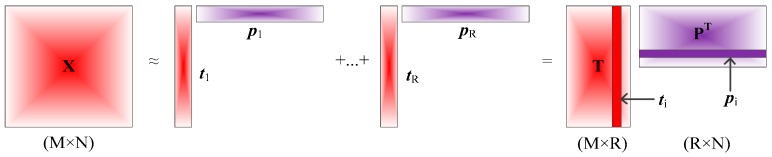
Matrix **X** decomposition of rank one (CP decomposition).

In the following step, we will apply one matrix decomposition technique, which is also depicted as a space projection from one space to another space, to extract the intrinsic features and restore the clean sensor signals. Actually, matrix decomposition is also known as a two-way tensor decomposition. In this manuscript, a popular tensor decomposition technique, which is called as CP (CANDECOMP/PARAFAC) decomposition [29], can be considered to be applied for matrix decomposition. The CP decomposition factorizes a matrix into a sum of component rank-one matrices, and can restore the cleaner sensor signals [29,30]. For example, the original sensors matrix **X** can be represented approximately as a sum of *R* matrices:

**X**≈**K**_1_ + **K**_2_ + **K**_3_ + ... + **K***_R_* (2)

where *R* is a positive integer, and **K***_i_* is *i*-th matrix of rank one, and the graphical representation is shown in Figure 1.

Meanwhile, the matrix **K***_i_* of rank one can also be denoted as outer products of two vectors, a score ***t****_i_*, a loading ***p****_i_*, and the mathematical formula is as follows:

(3) $$\begin{array}{ll} X & ≅\;t_{1}\circ p_{1}\;+\;t_{2}\circ p_{2}\;+\text{​​}\cdots \;+\;t_{R}\circ p_{R}\\ & =\;t_{1}p_{1}^{Τ}\;+\;t_{2}p_{2}^{Τ}\;+\;\cdots \;+\;t_{R}p_{R}^{Τ}\; \end{array}$$

where the symbol “∘” denotes outer product operator, both ***t****_i_* and ***p****_i_* are column vectors, and *T* denotes matrix or vector transpose.

Since iterative matrix decomposition is a robust procedure in CP decomposition, we adopt a known nonlinear iterative method [22,31] for calculating the score vector ***t*** and loading vector ***p***. The iterative process goes as follows:

1. Take some column vector ***x****_i_* as a starting vector ***t***: ***t***_start_ = ***x****_i_*
2. Normalize ***t***_start_ to length 1: ***t***_new_ = ***t***_start_/‖***t***_start_‖
3. Calculate a new loading vector ***p***: ***p*** = **X**^T^***t***
4. Normalize ***p*** to length 1: ***p***_new_ = ***p***_old_/‖***p***_old_‖
5. Calculate a new score vector ***t***: ***t*** = **X*p***
6. Normalize ***t*** to length 1: ***t***_new_ = ***t***_old_/‖***t***_old_‖
7. Check for convergence by comparing the new ***t*** vector with the previous ***t*** vector obtained in step 2. If convergence is apparent, go to step 8. If there is no convergence, continue with step 3.
8. Compute the residual matrix **E**: **E** = **X** − ***tp***^T^, and the value of *R* is determined by the norm of the residual matrix **E** (when ‖**E**‖ > 0.1, go on).

To realize the above iterative procedure, the detailed pseudo code has been summarized in Algorithm 1 as follows.
**Algorithm 1.** Pseudo Code for Iterative Matrix Decomposition.Inputs: MEG sensors space matrix $X\in {ℜ}^{N\times M}$Process: 1. For *i* = 1,…,*R*  Randomly initialize ***t***  ‖t‖→1, $\hat{t}=2t$  While $\left\|\hat{t}-t\right\|>\mathrm{eps}$   $\hat{t}=t$   $p=X^{T}t$   ‖p‖→1   t=Xp   ‖t‖→1  End  Extract the score and loading vector ***t***, ***p***  Deflate ***X*** matrix: $X=X-tp^{T}$ End 2. $T=[t_{1},\;t_{2},\;...,\;t_{R}],\;P=[p_{1},\;p_{2},\;...,\;p_{R}]$ 3. Reconstruct the sensors matrix $\hat{X}=TP^{T}$Outputs: Score matrix **T**, loading matrix **P**, and reconstruction matrix $\hat{X}$.

Thus, cleaner MEG sensor signals via signal reconstruction are obtained using the above-mentioned matrix decomposition algorithm, and can be used to estimate the weight vector via vector beamforming technique.

### 2.2. Vector Beamforming Estimator

The vector beamforming technique is implemented as a weighted sum of the surface recordings at different sensors during one time series. The weight vector is optimized by minimizing the filter output power subject to a linear constraint. In practice, data covariance **C**(***x***) is unknown and must be estimated firstly from the available sensors data in the LCMV method. In a general way, we chose a second-order statistic for sample to estimate the population covariance under the inherent assumption of a Gaussian distribution, and the approximate estimation is shown in Equation (4):

(4) $$\hat{C}(x)=\frac{1}{M-1}\sum_{i=1}^{M}\left(x_{i}-\bar{x}\right){\left(x_{i}-\bar{x}\right)}^{T}$$

where:

(5) $$\bar{x}=\frac{1}{M}\sum_{i=1}^{M}x_{i}$$

denotes the sample mean. Since the minimum-variance estimator is very sensitive to errors in estimating the sensors data covariance matrix, we substitute the new reconstructed matrix $\hat{X}$ into Equation (4) to estimate the covariance matrix.

Then, we apply an objective function according to vector beamforming estimator [15], and its mathematical formulation is:

(6) $$\underset{W}{\min}\;[E({\left\|W\hat{X}\right\|}^{2})]\;\mathrm{with}\;WL(r_{0})=1$$

where E(⋅) denotes the expectation value function, **W** is the weight matrix, and ***r***_0_ denotes one position of the grid point in all partitioned voxels. For obtaining the optimum solution of weight matrix **W**, an optimization algorithm of minimizing the interference (MinInf) [32] is employed for passing the activity at position ***r*** = ***r***_0_ with unit gain, while inhibiting contributions from all other sources. The optimal solution can be computed by minimizing the corresponding Lagrange function as:

(7) $$W\left(r\right)={\left[L^{T}\left(r\right){\hat{C}}^{-1}\left(x\right)L\left(r\right)\right]}^{-1}L^{T}\left(r\right){\hat{C}}^{-1}\left(x\right)$$

where **W**(**r**) indicates the projection matrix at the grid location **r**, ${\left(\cdot \right)}^{-1}$ denotes the inverse operator, and $\hat{C}(x)$ is the covariance matrix of random variables based on row vectors of the reconstructed sensors matrix $\hat{X}$. Additionally, the power value at each grid point, which can also be seen as the dipole moment, is expressed as:

(8) $$\mathrm{Pow}\left(r\right)=\mathrm{tr}\left\{\;{\left[L^{T}\left(r\right){\hat{C}}^{-1}\left(x\right)L\left(r\right)\right]}^{-1}\right\}$$

where Pow(**r**) indicates the power value at the grid location **r** and tr{⋅} denotes the trace of the matrix in braces. Therefore, the grid point of the power maximum, based on all grid points under the cortex constraint, would be selected as the location of the deep source.

### 2.3. Data Preprocessing and Artifact Rejection

In this manuscript, three primary toolboxes including Matlab R2014a (The MathWorks Inc., Natick, MA, USA), SPM8 [33], and FieldTrip [34] were used jointly for MEG data analysis. The MEG signal with continuous data, which would not be divided into many segments, was filtered by a band-pass filter of 0.5–60 Hz, notch-filtered at 50 Hz, and detrended via removing the linear trend from the data. Then noisy MEG channels were detected by the module of manual artifact rejection, and the bad channels were repaired automatically using a spline interpolation algorithm. Taking into account as much as possible to evaluate the signal-to-noise ratio (SNR), independent component analysis (ICA) and principal component analysis (PCA) were used for removing the artifacts related to hearbeats, muscles and eye blinks. We hoped that the interested signal containing spike was very clean. In the next step, the detailed procedure of source localization will be addressed.

### 2.4. Source Localization Algorithm

To further study the simulation and real data, we give a deep source localization procedure (see Algorithm 2). First, the individual anatomical magnetic resonance images (MRI; T1-weighted) and the digitised head shapes were co-registered to the MEG coordinate system using anatomical landmarks via an iterative closest point (ICP) algorithm [35]. Then, we added the cortex constraint in the source space in the proposed localization algorithm. To compute the forward solution, a realistically-shaped single-shell approximation was used for constructing a volume conduction model based on the implementation from Nolte [36].

Thus, the new procedure would be summarized into an algorithm as follows. It is assumed that data preprocessing and artifact rejection have been accomplished for the MEG data and individual MRI.
**Algorithm 2.** Deep Source Localization Procedure.**Forward solution:** 1. Construct the volume conduction model **V** based on a single shell approximation under the cortex constraint. 2. Calculate the lead field matrices **L** under the cortex constraint. 3. Denote the lead field matrix **L**_i_ corresponding to the i-th grid point.**MEG sensor space decomposition:** 1. Pick out the k-th data segment **X**^k^, of note, epilepsy patients data need to be contained the spike, and the time window width is about 200ms. 2. Decompose the data matrix **X**^k^ as score components **T**^k^ and loading matrix **P**^k^ using the *Algorithm 1*. 3. Reconstruct the sensors matrix ${\hat{X}}^{k}$.**Inverse solution:** 1. Estimate the covariance matrix ${\hat{C}}^{k}$ using the Equation (4). 2. Obtain the optimized weight matrix **W**^k^(**r**) at the grid location **r** based on the Equation (7). 3. Calculate the power value Pow^k^(**r**) at the grid location **r** via the Equation (8).**Localization results display:** 1. Select those grid points corresponding to the larger power values. 2. Visualize the result on the individual MRI using FieldTrip toolbox.

In the following, we will illustrate the effectiveness of the above algorithm both in simulation data and in real epilepsy patients.

### 2.5. SPS Algorithm

In this manuscript, the Signal Processor Software (SPS) method is specifically referred to as the data processing software of MEG equipment produced by Elekta Neuromag Oy (Helsinki, Finland), and the version number was 2.94. At present, although many so-called improved source localization methods have been proposed, the SPS is effective in localizing epileptogenic foci, and has been widely accepted by many neurologists in clinical application. The SPS method includes a series of MEG or EEG data analysis procedures, such as denoising, preprocessing, spectral analysis in time and space, construction of individual head models, solving inverse problem, and visualizing the results, etc. [27]. It is worth noting that the MaxFilter is used to remove noise and artifacts including those generated by noise sources inside the magnetic shielded room. The MaxFilter is also a kind of signal space separation in a spatio-temporal approach, and has been shown to outperform some classical filters like SSP and SSS [19]. Moreover, the dipolefitting method will be used to solve the inverse problem, and it is very similar to the Equivalent Current Dipole (ECD) method. Except for filtering and solving inverse problem, the rest of the steps are similar to the previous Algorithm 2. In short, the SPS method is closed in the MEG devices produced by Elekta Neuromag, and has been a relatively optimized method for localizing the epileptogenic foci in clinical application. Of note, we can only accomplish all SPS operations on the specified equipment, in which all the processes are strictly complied with the instructions provided by Elekta Neuromag Oy, and are completed by an experienced clinical MEG data analyst.

## 3. Experimental Results

### 3.1. Simulated data Description and Results

In this section, we took experiments on the simulated sensors data as follows. First, the simulated MEG sensor signals were generated by using the function ft_dipolesimulation in the FieldTrip toolbox from a given source. Second, we applied the realistic head model, which was a single shell model by using the magnetic resonance imaging (MRI) scan data of the first epilepsy patient in the Section 3.2. Third, we partitioned the inside brain space under the cortex constraint into a 3D grid with a resolution of 1 mm, and yielded a grid with 3704 points for the realistic head model. Then, we opted some typical different locations including superficial sources and deep sources to verify the localization accuracy of the proposed algorithm. Without loss of generality, those locations were from the principal areas of the cortex, such as the frontal, temporal, parietal and occipital lobes, of which temporal lobes were divided into lateral and mesial temporal lobes. In the Neuromag coordinate system, we put six non-null sources at location r represented from r_1_ to r_6_, where {r} was equal to {(59, 43, 70), (67, 11, 30), (67, −29, 86), (59, −53, 54), (35, 11, 38), (−29, 11, 38)} mm, and these locations corresponded to the approximate centers of those regions, which were right frontal, right lateral temporal, right parietal, right occipital, right hippocampus and left hippocampus respectively. Among these locations, the last two locations denoted deep sources, while the others were superficial sources. For the realistic head model, we selected a oscillatory source at location ***r*** with time-course:

(9) m(t)= Ω cos(2πft+φ)

where Ω denoted amplitude, ***f*** was frequency and *φ* denoted phase, and these parameters would be randomly adopted, for example as Ω = 2, ***f*** = 15, *φ* = 10 in this paper. In addition, the white Gaussian noise (WGN) was added into the time-course of the real signal, and the simulated sensor signal was generated in this manner. Herein, the index about the signal-to-noise ratio (SNR) was chosen for describing different noise levels, and the definition was expressed in the following formula:

(10) $${\mathrm{SNR}}_{\mathrm{dB}}=\;10\log_{10}\;\left(\frac{P_{A}}{P_{B}}\right)\;=\;10\log_{10}\;\left(\frac{{\left\|A\right\|}_{F}^{2}}{{\left\|B\right\|}_{F}^{2}}\right)$$

where P_A_ denotes the power of the simulated sensor signal, P_B_ expresses the power of background noise and ||**A**||_F_ means the Frobenius norm of matrix **A**. To illustrate the robustness of the source localization algorithm under the different background noise levels, we gave 12 different SNR levels in total, and randomly realized 100 WGNs on each noise level.

**Figure 2:**
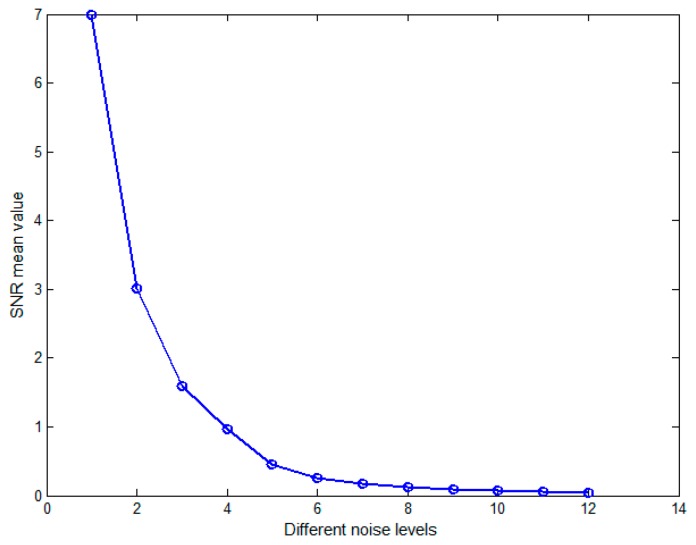
The SNR mean value of the simulated sensor data at twelve different noise levels.

**Figure 3:**
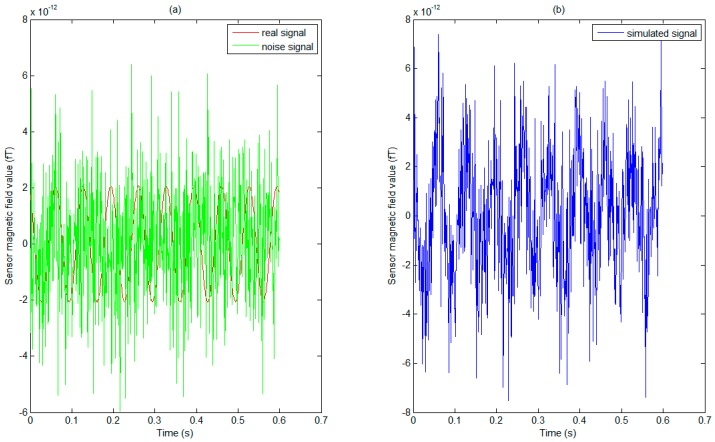
The real signal, the noise signal and the simulation signal were described through one of the sensors signal. (**a**) The red waveform represents the real signal, and the green signal represents noise under a 600 ms time window; (**b**) The simulation signal was composed of a real signal and an additive noise signal, in which the real signal is a cosine oscillation, and the noise signal is Gaussian white noise (SNR = 0.459).

Figure 2 showed the SNR mean value of simulated sensor data on twelve different noise levels, and SNR values from the maximum 6.990 down to the minimum 0.043. Then, the simulated sensor signal was generated by adding WGN, and one of the sensors signal was displayed in Figure 3, which depicted a real signal, noise signal and simulated sensor signal respectively under the SNR level of 0.459.

**Figure 4:**
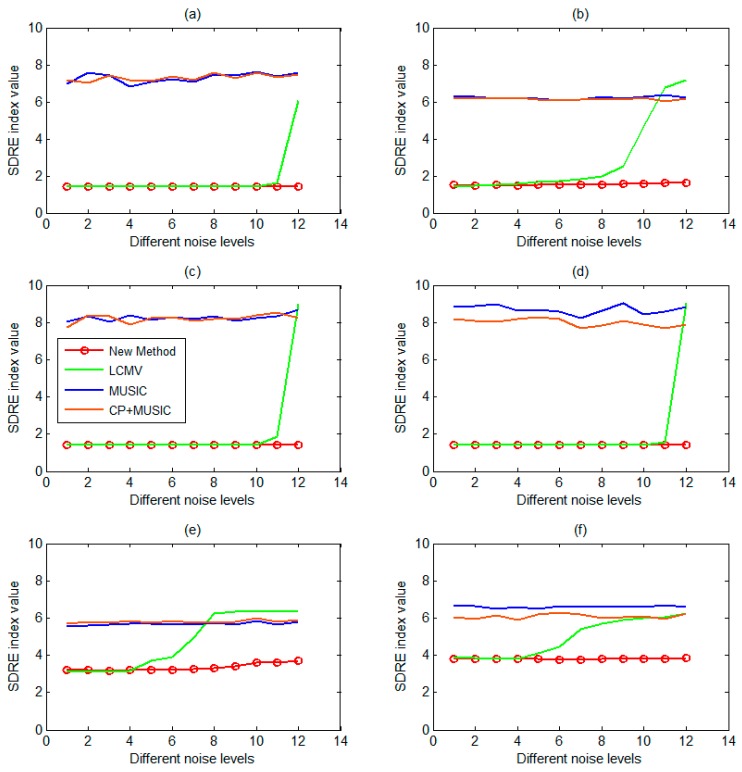
The source localization results of four different algorithms (the proposed new method, LCMV, MUSIC, and CP+MUSIC) were compared based on six different locations sources, in which the horizontal coordinates of each subgraph represented different noise levels, and the ordinates represented the mean of different SDRE values with 100 random results at the same noise level. (**a**) The mean of SDRE values varies with different noise levels based on the real source located in the right frontal lobe; (**b**) The mean of SDRE values varies with different noise levels based on the real source located in the right lateral temporal lobe; (**c**) The mean of SDRE values varies with different noise levels based on the real source located in the right parietal lobe; (**d**) The mean of SDRE values varies with different noise levels based on the real source located in the right occipital lobe; (**e**) The mean of SDRE values varies with different noise levels based on the real source located in the right mesial temporal lobe (right hippocampus); (**f**) The mean of SDRE values varies with different noise levels based on the real source located in the left mesial temporal lobe (left hippocampus).

In the next step, the abovementioned Algorithm 2 (deep source localization procedure) would be used for localizing those sources from the simulated sensor signal. To describe the effectiveness of the proposed method, we compared the new method with two other commonly used beamforming methods that were LCMV and MUSIC. In addition, in order to describe the validity of the iterative CP matrix decomposition, we also combined the CP decomposition with the MUSIC method, and compared the Algorithm 2 with the results of this method. Of note, the LCMV and MUSIC method were operated by using the FieldTrip toolbox. Next, the Space Distance between Real source and Estimated source (SDRE) index was used to evaluate the source localization algorithm. Figure 4 shows that the new method proposed in this paper had the best spatial accuracy, and had little influence on the localization accuracy with the increase of noise. Especially, the new method had significantly improved the deep source localization, and the localization accuracy of the new method was almost the same as that of the LCMV method in the shallow sources case. Yet, LCMV was clearly more sensitive to noise. In addition, the iterative CP decomposition was not obvious for the improvement of MUSIC. We would describe the localization accuracy of the deep sources in detail through the subgraph (e) of Figure 4. When using the proposed new method, the SDRE value was around 3.2 when the SNR value was greater than 0.1 (noise level: 1~7). In addition, the difference between SDRE maximum and minimum using the proposed method and LCMV method was 1.13 and 3.26, respectively. Further, using the LCMV method, the SDRE value suddenly increased (a drop in spatial accuracy) when the SNR was below 0.1 (noise level: 8~12). Meanwhile, the SDRE value using MUSIC method was almost all around 5.6 at all SNR values (noise level: 1~12). Therefore, under the same conditions, the proposed new method is more effective in finding deep sources in the sense of spatial accuracy than the LCMV, MUSIC, and CP+MUSIC methods, and the proposed method is relatively less influenced by additive noises.

### 3.2. Patients and Data Description

Two experienced clinical epileptologists visually marked epileptic spikes respectively in MEG signals after the previous step and ruled out drowsiness. For describing the preliminary effectiveness of the proposed algorithm, we collected a total of 10 patients with medically refractory unilateral mTLE retrospectively, in which at least 15 spikes were found on each patient during interictal MEG data. Mesial TLE was diagnosed based on a comprehensive preoperative evaluation, including seizure history and semiology, neurologic examination, 3 Tesla MR imaging, scalp electro-encephalography, invasive electroencephalography and pathology. Those patients had already undergone a standard clinical presurgical evaluation including clinical seizure semiology, long-term video-EEG monitoring, high-resolution MRI, MEG, PET, and/or interictal SPECT, as well as neuropsychological testing. According to all of these examination results, the preoperative assessment conclusion was given by Beijing epilepsy center expert group members. Then, all of those patients also underwent anterior temporal lobectomy [37] for focal epilepsy at Xuan Wu Hospital Capital Medical University (XWHCMU) between January 2013 and January 2016, which included at least one year of postoperative follow-up. The follow-up results showed that all of those patients were free of disabling seizures (Engel class IA) after surgery. Informed consent for the study was obtained from all patients. The study was performed under a protocol approved by the medical ethics committee of the XWHCMU Committee.

*MRI evaluation.* Preoperative MRI was performed on a Siemens 3T MAGNETOM Verio instrument equipped with a 32-channel head coil (Siemens Healthcare, Erlangen, Germany). The standard MRI protocol included T1-weighted, T2-weighted, fluid-attenuated inversion recovery (FLAIR) axial, T2 and FLAIR oblique coronal, fast inversion recovery with myelin suppression, and three-dimensional (3D) gradient echo coronal T1 images with whole-brain coverage. An individual three dimensional T1-weighted MRI with spatial resolution of 0.52 mm × 0.52 mm × 0.52 mm (with slice interpolation) was used to create a single-layer realistic head model for each patient. Preoperative MRI scans were reviewed separately and results confirmed by two neuroradiologists specializing in epilepsy and blinded to the seizure focus.

*MEG acquisition.* The MEG recordings were acquired inside a magnetically-shielded room by, 306 channels in total, with a helmet-shaped whole-head system (VectorView, Elekta Neuromag Oy), comprising 102 locations at triplets including one magnetometer and two orthogonal planar gradiometers. For mTLE patients, continuous data were recorded at a sampling rate of 1000 Hz for the MEG signal, and the electrocardiography data was recorded simultaneously. Each recording consisted of six 10-min epochs while they were lying in a supine position with eyes closed and in a resting state. A three-dimensional digitizer, which was a Polhemus^TM^ system (Polhemus, Colchester, NH, USA), was used to determine the location based on anatomical fiducial points (nasion, bilateral preauricular points) for the following MRI-MEG co-registration. Through checking the uniform distribution of points as far as possible covering the whole scalp, the head shape of each patient was ascertained ensuring that the head of patient cannot be moved throughout whole procedure.

### 3.3. Epileptogenic Zone Localization Results

Clinical characteristics of the 10 patients were listed in Table 1, and those epilepsy patients were judged as mesial Temporal Lobe (TL) origination based on preoperative evaluation and postoperative follow-up. From the clinical experience, the localization results could be found in the mesial TL region, and may also be found in the lateral or extra TL region, because the discharge sources may spread from one location to another during a short time. In general, we cannot guarantee that each spike was generated by the mesial TL discharge for clinically-diagnosed patients with mTLE.

**Figure 5:**
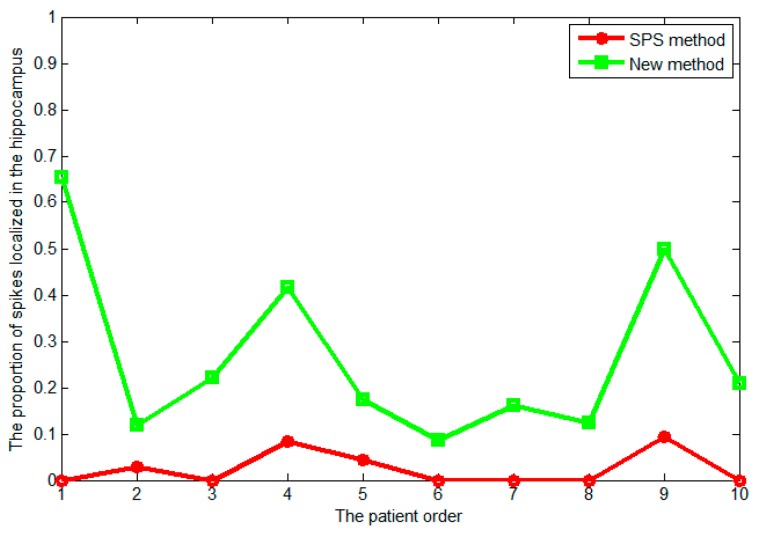
Comparison of the new method and SPS method on deep source localization results, in which the x-axis represents the patient sequence, and the y-axis represents the ratio of the number of spikes localized in the hippocampus to the total number of spikes on each patient.

In order to observe the effectiveness of the new method on deep source localization, all patients collected were clinically evaluated for mesial TL origination, and almost all spike localization sources were found in the lateral TL based on the SPS method. In addition, we selected an evaluating indicator to observe the effect of deep source localization, which was the ratio of the number of spikes localized in the hippocampus to the total number of spikes on each patient. Figure 5 shows that the ratio obtained by the newly-proposed method was clearly higher than that of the SPS method. It also showed that almost no spike localized in the mesial TL was found based on the SPS method, however, many spikes localized in the mesial TL were found based on the new method. Thus, it seems that the localization results using the new method are more consistent with clinical outcomes. Then, we compared the localization results of the SPS method with the new method by taking the results of the first two patients as an example, and all of the MEG source imaging results were shown from coronal and sagittal views as follows.

**Figure 6:**
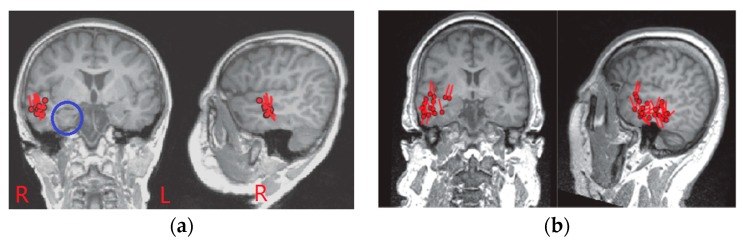
Magnetic source imaging results based on the SPS method based on all spikes. (**a**) Localization results for the first patient displayed on individual MRI from coronal and sagittal views, wherein the hippocampal region was denoted by a blue circle on coronal view, and the red mark “R” (or “L”) denoted the right (or left) side of the brain; (**b**) Localization results for the second patient displayed on individual MRI from coronal and sagittal views.

**Figure 7:**
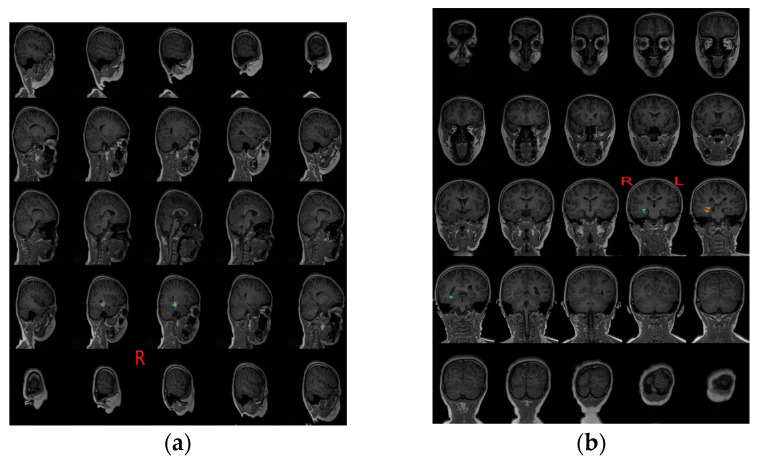
Source localization results for patient #1 based on a single spike using the proposed method. (**a**) Left panel represented a series of sagittal slices from the left to the right side of the brain, which corresponded to each line from right to left, and the red mark “R” denoted the right side of the brain, moreover, the darker color of the second and third slices in the last second rows indicated the estimated location of sources; (**b**) Right panel showed the same source location as the subgraph (**a**) through the coronal slices, in which the fourth and the fifth slice of the third line indicated the estimated location of sources, and the red mark “L” denoted the left side of the brain.

**Figure 8:**
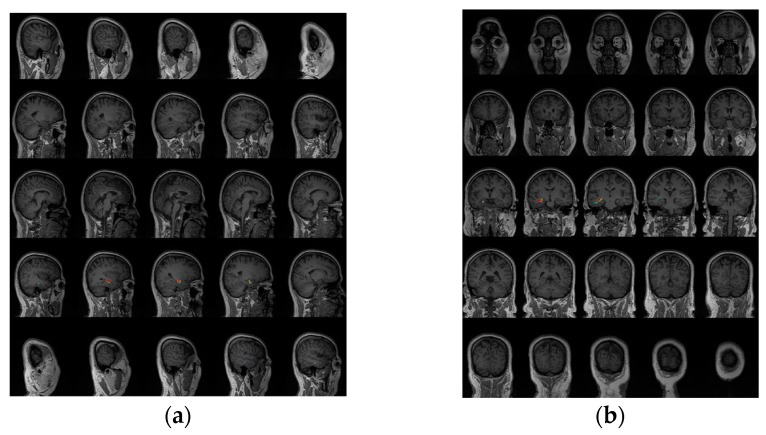
Source localization results for patient #2 based on a single spike using the proposed method, and the interpretation of subgraphs (**a**,**b**) was almost consistent with the subgraph interpretation of Figure 7.

First, Figure 6 gives the localization results using the SPS method, which were reviewed by clinical MEG neurologists, and these results showed that almost all of the dipoles were displayed in the lateral TL region. Second, the localization results using the proposed scheme were displayed on an individual MRI of the epilepsy patient. For clearly observing the deep regions around the hippocampus, we exhibited a figure including a total of 25 slices, five rows by five columns, from coronal and sagittal views respectively. Both Figure 7 and Figure 8 show that the localization results appeared in the hippocampus based on the proposed method, and these results were generated by a time series of MEG data including spike for the first patient and the second patient. In fact, each patient had some localization results of a certain number of spikes, appearing in the mesial TL, similar to these two images, and the ratio is shown in Figure 5. Of note, MRI examination results showed that the signal of the hippocampal region was normal for the second patient, and it probably illustrates that MRI examination generates false negative results. In addition, we observed that the source activity regions were also located in the anterior TL based on MEG using the two methods (the SPS method and the proposed method).

In a word, the above results showed that the proposed method was more reliable than the SPS method in mTLE patients, and the results of the proposed method were mostly consistent with results of the clinical comprehensive preoperative evaluation, surgical outcome, and postoperative follow-up (see Table 1). Therefore, the proposed method very likely has improved the classical beamforming methods (LCMV, MUSIC) and is superior over the SPS method in the accurate detection of deep sources in the preoperative evaluation of epileptic surgery.

## 4. Discussion and Conclusions

In previous studies, many studies have focused their attention on detecting surface sources in MEG measurements, and have found the source localization methods are effective and helpful for finding the epileptogenic zone of neocortical epilepsy [5,38,39,40,41]. However, the ability of detecting deep sources such as those in the mesial temporal lobe remains in question [7,8,9,42].

In this work, we proposed a modified beamforming approach for finding deep sources in the brain with MEG recordings. Since iterative spatiotemporal signal decomposition could characterize the intrinsic discriminant features for interpreting sensor signals, it was employed for reconstructing the sensor space. Then, the sensor covariance matrix was estimated under the new reconstructed space, and the beamforming using minimum variance with a linear constraint was applied for computing the solution for the inverse problem.

In MEG-simulated sensor data, the new method was superior to two classical beamforming methods (LCMV and MUSIC) in localization accuracy, which was least affected by noise, and especially in deep source localization, this method was more advantageous. Although the iterative CP decomposition was very effective for the LCMV improvement, the results of the MUSIC improvement was not obvious, which may be due to the essential difference between the principles of the LCMV and MUSIC methods.

According to clinical experience, we know that not all interictal spikes are produced by the mesial temporal lobe in mTLE patients, but at least part of the spikes may be produced by the mesial TL discharge. In clinical verification using mTLE patients, results of the proposed method seem to be more consistent with comprehensive results incorporating multi-modality neuroimages, clinical characteristics, and postoperative follow-up. Compared with the SPS method, the proposed method is more capable of detecting deep sources in the brain. This may help increase the clinical utility of MEG in preoperative evaluation in epileptic surgery with epileptogenic foci in deep brain regions. Yet, due to the limited number of mTLE patients, the proposed method only provides a pilot framework for accurately finding deep epileptogenic zone. To translate and generalize the proposed method in clinical application, a large number of patients would need to be recruited for further verification in the future.

## Figures and Tables

**Table 1:** Clinical characteristics of the patients.

Patient No.	Sex	Age/Seizure Duration (years)	MRI	MEG (SPS)	Spike Number	Preoperative Assessment	Surgical Procedure	Pathology
1	M	6/3	RHS	RT	26	RT	RATH	HS, FCD
2	M	41/18	Normal	RT, In	34	RT	RATH	HS, FCD
3	M	26/5	LHS	LT	18	LT	LATH+Am	HS, FCD
4	F	43/40	HRH	RT, In	15	RT	RATH+Am	HS, FCD
5	M	22/17	LHS	LT	23	LT	LATH+Am	HS, FCD
6	M	37/26	RHS	RT	35	RT	RATH+Am	HS, FCD
7	F	15/9	RHS	RT	37	RT	RATH	HS, FCD
8	F	27/11	RHS	RT	32	RT	RATH	HS, FCD
9	M	22/20	LHS	LT, Pa	32	LT	LATH	HS, FCD
10	F	39/13	BHS	RT, In	24	RT	RATH+Am	HS, FCD

M indicates male; F, female; Pa, parietal; In, insular; LT, left temporal; RT, right temporal; LHS, left hippocampal sclerosis; RHS, right hippocampal sclerosis; HRH, hyper T2 in right hippocampal; BHS, bilateral hippocampal sclerosis; LATH, left anterior temporal lobectomy including hippocampus; RATH, right anterior temporal lobectomy including hippocampus; Am, amygdala; FCD, focal cortical dysplasia.
